# Both levoglucosan kinase activity and transport capacity limit the utilization of levoglucosan in *Saccharomyces cerevisiae*

**DOI:** 10.1186/s13068-022-02195-x

**Published:** 2022-09-14

**Authors:** Mengdan Yang, Tiandi Wei, Kai Wang, Liqun Jiang, Dihao Zeng, Xinhua Sun, Weifeng Liu, Yu Shen

**Affiliations:** 1 State Key Laboratory of Microbial Technology, Institute of Microbial Technology, Shandong University, No. 72 Binhai Road, Qingdao, 266237 China; 2 Guangdong Engineering Laboratory of Biomass High-Value Utilization, Guangzhou Key Laboratory of Biomass Comprehensive Utilization, Guangdong Plant Fiber Comprehensive Utilization Engineering Technology Research and Development Center, Institute of Biological and Medical Engineering, Guangdong Academy of Sciences, Guangzhou, 510640 China

**Keywords:** *Saccharomyces cerevisiae*, Levoglucosan, Transporter, 1,6-Anhydro-N-acetylmuramic acid kinase, Gal2p

## Abstract

Manufacturing fuels and chemicals from cellulose materials is a promising strategy to achieve carbon neutralization goals. In addition to the commonly used enzymatic hydrolysis by cellulase, rapid pyrolysis is another way to degrade cellulose. The sugar obtained by fast pyrolysis is not glucose, but rather its isomer, levoglucosan (LG). Here, we revealed that both levoglucosan kinase activity and the transportation of levoglucosan are bottlenecks for LG utilization in *Saccharomyces cerevisiae*, a widely used cell factory. We revealed that among six heterologous proteins that had levoglucosan kinase activity, the 1,6-anhydro-N-acetylmuramic acid kinase from *Rhodotorula toruloides* was the best choice to construct levoglucosan-utilizing *S. cerevisiae* strain. Furthermore, we revealed that the amino acid residue Q341 and W455, which were located in the middle of the transport channel closer to the exit, are the sterically hindered barrier to levoglucosan transportation in Gal2p, a hexose transporter. The engineered yeast strain expressing the genes encoding the 1,6-anhydro-N-acetylmuramic acid kinase from *R. toruloides* and transporter mutant Gal2p^Q341A^ or Gal2p^W455A^ consumed ~ 4.2 g L^−1^ LG in 48 h, which is the fastest LG-utilizing *S. cerevisiae* strain to date.

**Supplementary Information:**

The online version contains supplementary material available at 10.1186/s13068-022-02195-x.

## Background

Lignocellulosic materials, which are an abundant and renewable resource, can be used instead of fossil-based resources to produce biofuels and chemicals, and are a promising alternative to reducing environmental pollution while ensuring energy security. Lignocellulosic materials are generally pretreated by acids or bases at high temperatures to unlock their crosslinks between cellulose, hemicellulose, and lignin [1–3]. Hemicelluloses are generally saccharified and dissolved during the pretreatment; however, cellulose remains solid and requires other saccharification processes in order for glucose to be obtained. Common saccharification processes consist of degrading cellulose by cellulase [4]. Pyrolysis, during which cellulose is treated with heat (300–600 °C) in a very short time, is another option to saccharify cellulose [5, 6]. The main sugar obtained by this fast pyrolysis process is levoglucosan (LG) [7, 8], which is an isomer of glucose and also known as 1,6-anhydro-β-d-glucopyranose [9].

**Fig. 1:**
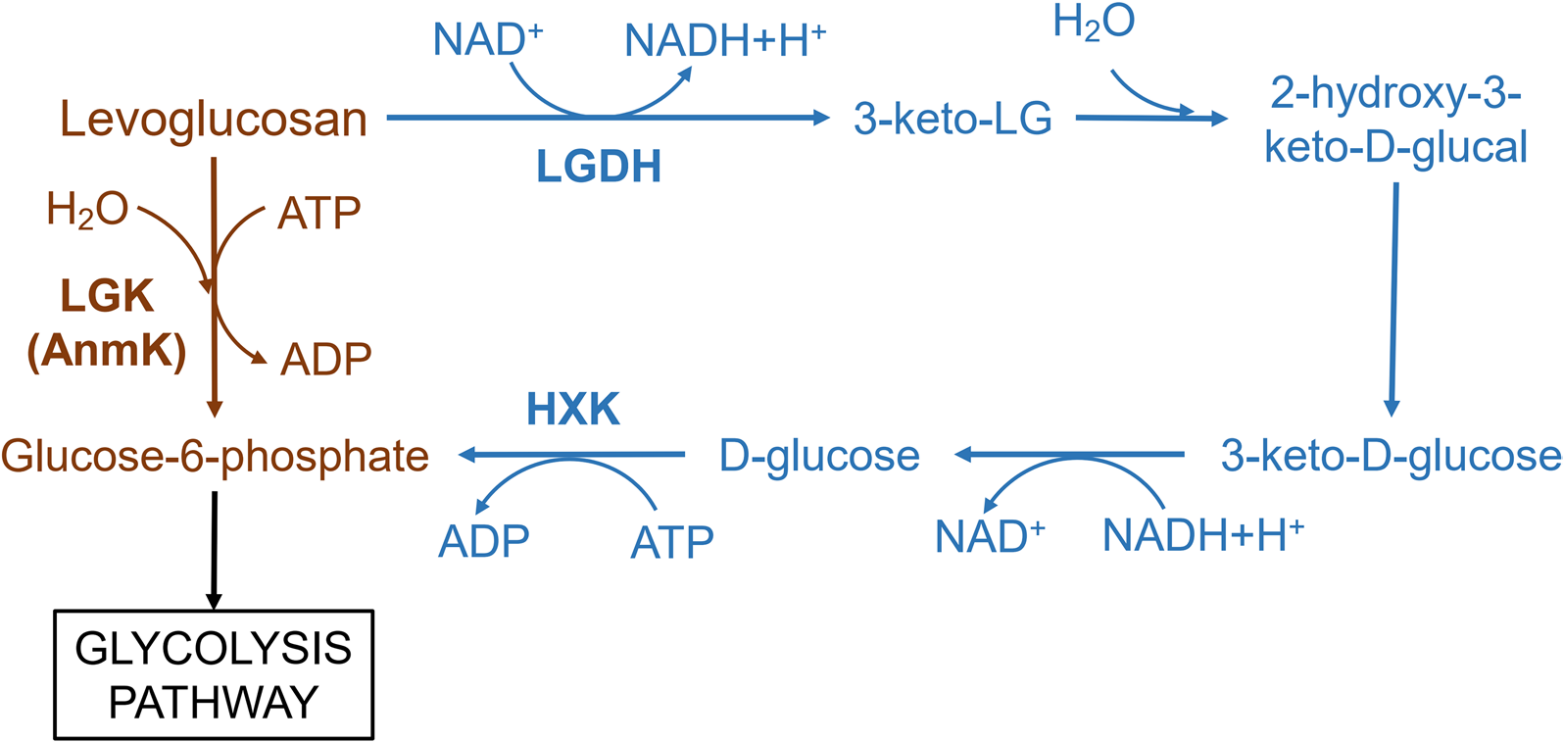
Metabolic pathways of levoglucosan in microorganisms. *LGK* levoglucosan kinase, *AnmK* 1,6-anhydro-N-acetylmuramic acid kinase, *LGDH* levoglucosan dehydrogenase, *HXK* hexokinase. Enzymes that catalyze the additional steps in this pathway have not been formally named. The pathway starting from LGK is shown in brown; while the pathway starting from LGDH is shown in blue

To date, two pathways for the metabolism of levoglucosan have been identified (Fig. 1) [10, 11]. First, levoglucosan is phosphorylated into glucose-6-phosphate by levoglucosan kinase (LGK) or 1,6-anhydro-N-acetylmuramic acid kinase (AnmK). Glucose-6-phosphate is then further metabolized through glycolysis pathway [12, 13]. Second, levoglucosan can be sequentially metabolized through oxidation, β-elimination, hydration, and reduction. However, the enzymes involved in this pathway are not well studied except for levoglucosan dehydrogenase (LGDH) [11, 14]. Some proteins with LGK activity have been reported, including proteins from *Rhodosporidium toruloides*, *Rhodotorula glutinis* [15], *Aspergillus terreus* [16], *Aspergillus niger* [17], and *Lipomyces starkeyi* [18].

*Saccharomyces cerevisiae* is a commonly used cell factory because of its high sugar tolerance, high fermentation rate, and vigorous growth [19]. However, *S. cerevisiae* cannot utilize levoglucosan, which was considered to be due to the absence of levoglucosan kinase. Although the lack of this one step seemed like a problem that could be easily remedied, very few works have constructed levoglucosan-utilizing *S. cerevisiae*. However, metabolic engineering work for the inclusion of levoglucosan kinase has been successful in *Escherichia coli* [20], *Corynebacterium glutamicum* [12], and *Rhodococcus jostii* [21]. As far as we know, only one study related to the expression of the LGK gene in *S. cerevisiae* has been published. The gene in this study was isolated from *Aspergillus niger*, and transformations obtained two strains that were able to grow on selective media using levoglucosan as the sole carbon source. However, no fermentation results were disclosed [22].

Besides the activity of enzyme in the metabolic pathway, nutrient use efficiency also depends on the cell's ability to uptake the nutrient efficiently from environment. *S. cerevisiae* harbors a complex family of hexose transporters that encompasses 18 carrier proteins (Hxt1–17 and Gal2) with different characteristics. Common monosaccharides, such as glucose, mannose, galactose, xylose, and so on, are transported through this system [23]. Here, we first evaluated six heterologous proteins, all of which claimed to have LG activity in *S. cerevisiae* and revealed that among them, the Anmk gene of *Rhodotorula toruloides* was the best choice to construct a strain of LG-utilizing *S. cerevisiae*. However, the LG consumption rate of strain expressing this Anmk was still low. We then investigated the effect of transport on LG utilization. In a 3D structure model, ten amino acid residues of the sugar transporter Gal2p with a distance of  < 4 Å from levoglucosan were changed to alanine, and it was found that mutations of Q341A or W455A in Gal2p lead to a ~ tenfold increase in levoglucosan consumption. This result strongly suggested that the transport of levoglucosan was a serious limiting step for the utilization of LG. Furthermore, we analyzed the location of Q341 and W455 in the structure model of Gal2p in conjunction with the docking of LG, and indicate that the mutations of Q341A or W455A removed the sterically hindering barrier in the way of levoglucosan’s entry into the yeast cell.

## Materials and methods

### Strains and plasmids

To evaluate the function of LGKs and AnmKs, the codons of the optimized genes were synthesized by GENEWIZ Biotechnology Co., LTD (Suzhou, China) according to the sequence information supplied by NCBI (https://www.ncbi.nlm.nih.gov/) and individually ligated into vector pJFE3 [24]. The recombinant plasmids were then, respectively, transformed into *S. cerevisiae* strain CEN.PK113-5D [25] using the LiAc/ss-DNA/PEG transformation method [26].

The AnmK gene from *Rhodotorula toruloides* was also cloned into plasmid pIYC04 [27], resulting in the plasmid pIYC04-Rho. pIYC04-Rho was transformed into *S. cerevisiae* strain EBY.VW4000, which lacked all of the 18 native hexose transporters [28]. The resulting strain YLGR000 was used as the chassis cell to evaluate the function of transporter Gal2p and its mutants. The gene *GAL2* was amplified from CEN.PK113-5D. Fusion PCR was used to obtain the mutants of *GAL2*. The mutants then were cloned into pJFE3 and transformed into YLGR000. YLGR000 transformed with empty pJFE3 and pJFE3-GAL2^WT^ were used as references. All of the plasmids and strains used in this work are listed in Table 1, and all of the primers are in Additional file 1: Table S1.

**Table 1:** Strains and plasmids used in this study

Strains and plasmids	Genotype	Source
CEN.PK 113-5D	MATa; ura3-53	[25]
CEN-pJFE3	CEN.PK 113-5D derivative; pJFE3	This work
CEN-Rho	CEN.PK 113-5D derivative; pJFE3-Rho	This work
CEN-Sch	CEN.PK 113-5D derivative; pJFE3- Sch	This work
CEN-Mey	CEN.PK 113-5D derivative; pJFE3- Mey	This work
CEN-Lip	CEN.PK 113-5D derivative; pJFE3- Lip	This work
CEN-Asp	CEN.PK 113-5D derivative; pJFE3- Asp	This work
CEN-Koc	CEN.PK 113-5D derivative; pJFE3- Koc	This work
EBY.VW4000	*MATα leu2-3,112 ura3-52 trp1-289 his3-Δ1 Mal2-8c SUC2 hxt17Δ hxt13Δ::loxP hxt15Δ::loxP hxt16Δ::loxP hxt14Δ::loxP hxt12Δ::loxP hxt9Δ::loxP hxt11Δ::loxP hxt10Δ::loxP hxt8Δ::loxP hxt514::loxP hxt2Δ::loxP hxt367Δ::loxP gal2Δ stl1Δ::loxP agt1Δ::loxP ydl247wΔ::loxP yjr160cΔ::loxP*	[28]
EBY-pIYC04	EBY.VW4000 derivative; pIYC04	This work
EBY-pIYC04-pJFE3	EBY-pIYC04 derivative; pJFE3	This work
YLGR000	EBY.VW4000 derivative; pIYC04-Rho	This work
YLGR00P	YLGR00 derivative; pJFE3	This work
YLGR00G	YLGR00 derivative; pJFE3-GAL2^WT^	This work
YLGR085	YLGR00 derivative; pJFE3-GAL2^F85A^	This work
YLGR215	YLGR00 derivative; pJFE3-GAL2^Q215A^	This work
YLGR218	YLGR00 derivative; pJFE3-GAL2^I218A^	This work
YLGR341	YLGR00 derivative; pJFE3-GAL2^Q341A^	This work
YLGR342	YLGR00 derivative; pJFE3-GAL2^Q342A^	This work
YLGR346	YLGR00 derivative; pJFE3-GAL2^N346A^	This work
YLGR347	YLGR00 derivative; pJFE3-GAL2^N347A^	This work
YLGR350	YLGR00 derivative; pJFE3-GAL2^F350A^	This work
YLGR446	YLGR00 derivative; pJFE3-GAL2^Y446A^	This work
YLGR455	YLGR00 derivative; pJFE3-GAL2^W455A^	This work
YLGR2M	YLGR00 derivative; pJFE3-GAL2 ^Q341A W455A^	This work
pJFE3	2 μ expression vector with *URA3* marker, *TEF1* promoter, *PGK1* terminator	[24]
pJFE3-Rho	Coding gene of 1,6-anhydro-N-acetylmuramic acid kinase from *Rhodotorula toruloides* cloned into pJFE3	This work
pJFE3- Sch	Coding gene of 1,6-anhydro-N-acetylmuramic acid kinase from *Scheffersomyces stipites* cloned into pJFE3	This work
pJFE3- Mey	Coding gene of 1,6-anhydro-N-acetylmuramic acid kinase from *Meyerozyma guilliermondii* cloned into pJFE3	This work
pJFE3- Lip	Coding gene of levoglucosan kinase from *Lipomyces starkeyi* cloned into pJFE3	This work
pJFE3- Koc	Coding gene of levoglucosan kinase from *Kockovaella imperatae* cloned into pJFE3	This work
pJFE3- Asp	Coding gene of 1,6-anhydro-N-acetylmuramic acid kinase from *Aspergillus niger* cloned into pJFE3	This work
pIYC04	Yeast 2μ plasmid, *PGK1p-CYC1t*, *TEF1p-ADHt*, *HIS3* marke	[27]
pIYC04-Rho	Coding gene of 1,6-anhydro-N-acetylmuramic acid kinase from *Rhodotorula toruloides* cloned into pIYC04	This work
pIYC04-Sch	Coding gene of 1,6-anhydro-N-acetylmuramic acid kinase from *Scheffersomyces stipites* cloned into pIYC04	This work
pJFE3-GAL2^WT^	pJFE3-*TEF1p-GAL2-PGK1t*	This work
pJFE3-GAL2^F85A^	pJFE3-*TEF1p-GAL2*^*F85A*^*-PGK1t*	This work
pJFE3-GAL2^Q215A^	pJFE3-*TEF1p-GAL2*^*Q215A*^*-PGK1t*	This work
pJFE3-GAL2^I218A^	pJFE3-*TEF1p-GAL2*^*I218A*^*-PGK1t*	This work
pJFE3-GAL2^Q341A^	pJFE3-*TEF1p-GAL2*^*Q341A*^*-PGK1t*	This work
pJFE3-GAL2^Q342A^	pJFE3-*TEF1p-GAL2*^*Q342A*^*-PGK1t*	This work
pJFE3-GAL2^N346A^	pJFE3-*TEF1p-GAL2*^*N346A*^*-PGK1t*	This work
pJFE3-GAL2^N347A^	pJFE3-*TEF1p-GAL2*^*N347A*^*-PGK1t*	This work
pJFE3-GAL2^F350A^	pJFE3-*TEF1p-GAL2*^*F350A*^*-PGK1t*	This work
pJFE3-GAL2^Y446A^	pJFE3-*TEF1p-GAL2*^*Y446A*^*-PGK1t*	This work
pJFE3-GAL2^W455A^	pJFE3-*TEF1p-GAL2*^*W455A*^*-PGK1t*	This work
pJFE3-GAL2^Q341AW455A^	pJFE3-*TEF1p-GAL2 *^*Q341A W455A*^*-PGK1t*	This work

### Media and cultivation

CEN.PK113-5D was cultured in YPD medium (20 g L^−1^ tryptone, 10 g L^−1^ yeast extract, and 20 g L^−1^ glucose). CEN.PK113-5D transformed with plasmid pJEF3 or pJEF3 derived plasmids were cultured in SC-URA medium (1.7 g L^−1^ yeast nitrogen base, 5 g L^−1^ ammonium sulfate, 0.77 g L^−1^ CSM-URA) with 20 g L^−1^ glucose or 5 g L^−1^ levoglucosan as the carbon source. EBY.VW400 was cultured in YPM medium (20 g L^−1^ tryptone, 10 g L^−1^ yeast extract, and 20 g L^−1^ maltose). EBY.VW4000 transformed with plasmid pIYC04 or pIYC04-Rho were cultured in SC-HIS medium (1.7 g L^−1^ yeast nitrogen base, 5 g L^−1^ ammonium sulfate, 0.77 g L^−1^ CSM-HIS) with 20 g L^−1^ maltose or 5 g L^−1^ levoglucosan as carbon source. EBY.VW4000 transformed with plasmids pIYC04-Rho and pJEF3 or pJEF3 derived plasmids were cultured in SC-URA-HIS medium (1.7 g L^−1^ yeast nitrogen base, 5 g L^−1^ ammonium sulfate, 0.75 g L^−1^ CSM-HIS-URA) with 20 g L^−1^ maltose or 5 g L^−1^ levoglucosan as carbon source. All cells were cultured at 30 °C, with shaking at 200 rpm.

### Spot dilution growth assay

Single colonies were cultured in SC-URA medium supplemented with glucose as the carbon source for 12 h, and then transferred to fresh media when the OD_600_ reached 0.2 and cultured for another 12 h. These pre-cultured cells were collected and washed twice with sterile water and resuspended in ddH_2_O to an OD_600_ of ~ 1. Tenfold serial dilutions were performed, and 2 μL of each dilution was spotted onto the SC-URA plate with 5 g L^−1^ levoglucosan as carbon source. Then the plates were cultured at 30 °C until observable colonies were formed.

### Enzyme activity assays of LGK

Single colonies were cultured in 3 mL SC-URA medium supplemented with glucose as carbon source for 24 h, then transferred to 20 mL fresh media and cultured for 12 h. The cells were collected by centrifugation and resuspended in 40 mL fresh media with an initial OD_600_ of 1.0 and cultured for another 6–8 h to harvest at mid-log phase. Cell-free extracts were prepared using a homogenizer (Bertin, Precellys 24, France). The total protein in the cell-free extracts was determined by a BCA protein assay reagent kit (Beyotime, Shanghai, China).

Enzyme activity assays were performed according to the method detailed in a previous report. Briefly, the formation of NADPH associated with the reaction catalyzed by glucose-6-phosphate dehydrogenase was measured [21]. Each 1 µL reaction contained 50 mM Tris–HCL (pH 9.0), 75 mM levoglucosan, 10 mM MgCl_2_, 2 mM ATP, 0.2 mM NADP, 50 μL cell-free extract, and 1U glucose-6-phosphate dehydrogenase. The reaction was started with the addition of levoglucosan, and the absorbance at 340 nm was measured using a spectrophotometer (Presee, TU-1810, Beijing, Chian). One unit of enzyme activity was defined as the amount of enzyme which generated 1 nmol of NADPH per minute at 30 °C [15].

### Intracellular accumulation of levoglucosan

Intracellular accumulation of levoglucosan was characterized using a previously described method that is normally used to characterize the intracellular accumulation of other sugars [29, 30]. Single colonies were cultured in YPD or YPM medium at 30 °C with shaking at 200 rpm for 12 h and transferred into 20 mL fresh medium for another 12 h cultivation. The cells were collected and washed with ddH_2_O to be used as seed cells. The seed cells were resuspended in 30 mL YP medium supplied with 5 g L^−1^ levoglucosan to an initial OD_600_ of 1.0. The resuspended cells were incubated at 30 °C and 10 mL samples were taken at 30 min, 60 min, and 120 min. Immediately after the samples were collected, they were quickly washed twice with ice-cold ddH_2_O, then resuspended in 3 mL ddH_2_O and placed in 37 °C overnight to extract the intracellular levoglucosan.

### Homologous modeling of Gal2p and levoglucosan

The putative homology model of the transporter Gal2p was analyzed using the software SWISS-MOEDL (https://swissmodel.expasy.org/) using the crystal structure of transport XylEp in *Escherichia coli* [31] as a template. The software AUTODOCK v4.2 was used to analyze the molecular docking simulation between Gal2p and levoglucosan. The most likely docking position was determined according to the minimum free energy principle.

### Growth measurement and batch fermentation

Seed cultures of strains were prepared in YPM medium as described in ‘‘Intracellular accumulation of levoglucosan” and resuspended in SC-URA-HIS medium supplied with 5 g L^−1^ levoglucosan with an initial OD_600_ of 1.0. Then, 800 μL of the cell suspension was transferred to a 48-well plate, and the growth of strain was measured in a microplate reader (BioTek, Synergy HTX, USA). The seed culture of all the strains were prepared in YPM medium as described in ‘‘Intracellular accumulation of levoglucosan” and transferred into the 20 mL SC-URA-HIS medium supplied with 5 g L^−1^ levoglucosan as the carbon source. The initial OD_600_ was 1.0, and the fermentation was performed in 50-mL shake flasks at 30 °C with a shake speed of 200 rpm. The OD_600_ was measured with spectrophotometer (Eppendorf, BioPhotometer D30, Germany). The maximum specific growth rates (μmax) are the linear regression coefficients of the ln OD_600_ versus time during the exponential growth phase [32].

### Analysis of levoglucosan and metabolites

The concentration of levoglucosan and its metabolites were measured by HPLC using a Prominence LC-20A (Shimadzu, Japan) equipped with the refractive index detector RID-10A (Shimadzu, Japan) and Aminex HPX-87H ion exchange column (Bio-Rad, Hercules, USA). The mobile phase was 5 mM H_2_SO_4_, the flow rate was 0.6 mL min^−1^, and the temperature of the column was 45 °C. The levoglucosan transport capacity was defined as mg levoglucosan per g dry cell weight (DCW).

## Results and discussion

### Screening the AnmK or LGK that can actively express in *S. cerevisiae*

To build the levoglucosan metabolic pathway in *S. cerevisiae*, six genes encoding AnmK or LGK were synthesized and their sequences were optimized to use the codons preferred by *S. cerevisiae* (sequences are listed in Additional file 1). The protein sequences of AnmKs were obtained from *Aspergillus niger* (GenBank: CAK44911.1), *Meyerozyma guilliermondii* (GenBank: EDK40502.2), *Rhodotorula toruloides* (GenBank: CDR43051.1), and *Scheffersomyces stipitis* (GenBank: ABN66269.2); the protein sequences of LGKs were obtained from *Kockovaella imperatae* (GenBank: XM_022013599.1) and *Lipomyces starkeyi* (GenBank: ACE79748.1). Their evolutionary relationships are shown in a phylogenetic tree (Additional file 1: Fig. S1). The six genes were cloned into a 2μ plasmid vector pJFE3 to generate the plasmids pJFE3-Rho, pJFE3-Sch, pJFE3-Mey, pJFE3-Lip, pJFE3-Koc, and pJFE3-Asp. Then these recombinant plasmids were, respectively, introduced into *S. cerevisiae* strain CEN.PK113-5D, resulting in recombinant strains CEN-Asp, CEN-Mey, CEN-Rho, CEN-Sch, CEN-Koc, and CEN-Lip. CEN.PK113-5D with the empty vector pJFE3, CEN-pJFE3, was used as the control.

**Fig. 2:**
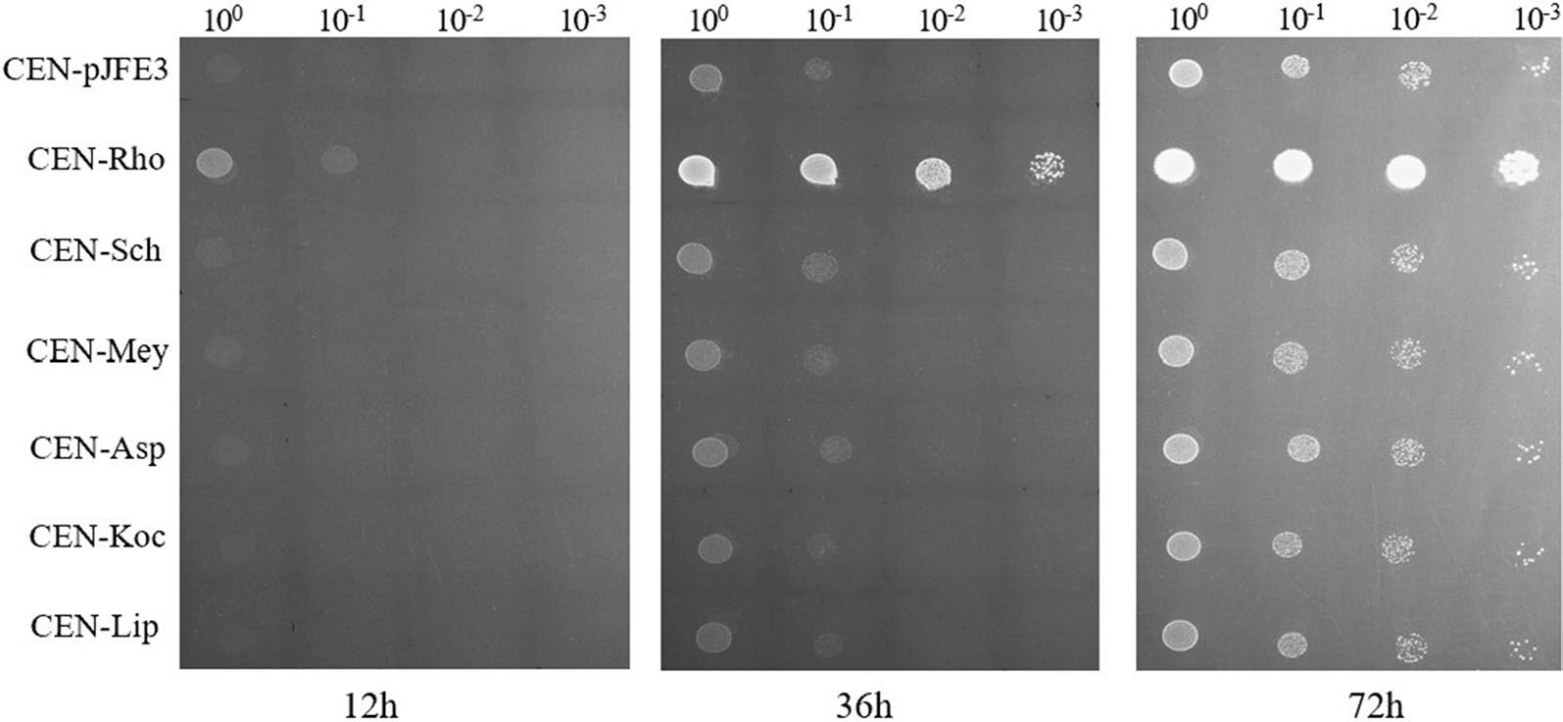
Growth of recombinant strains of *S. cerevisiae* on SC-URA medium supplemented with 5 g L^−1^ levoglucosan as the sole carbon source. Strains were pre-cultured overnight, transferred to fresh media and cultured for another 12 h. Cells were harvested and resuspended by sterile ddH_2_O until the OD_600_ of the suspension reached 1.0, and then a series of tenfold dilutions were spotted onto SC-URA media plates supplemented with 5 g L^−1^ levoglucosan

The recombinant strains were spotted onto a plate with SC-URA medium supplemented with 5 g L^−1^ levoglucosan as the carbon source to determine their growth capacity in levoglucosan. The result (Fig. 2) showed that the recombinant strain CEN-Rho, which expressed the AnmK of *R. toruloides*, grew much better than the other strains. Furthermore, the enzyme activity assay revealed that the levoglucosan kinase activity in the crude cell extracts of strain CEN-Rho was 10.65 ± 1.78 U/mg of protein, while the levoglucosan kinase activity in the crude cell extracts of strains CEN-Asp, CEN-Mey, CEN-Sch, CEN-Koc, CEN-Lip, and CEN-pJFE3 were 1.89 ± 0.97, 1.76 ± 1.02, 2.98 ± 0.86, 1.78 ± 0.93, 1.67 ± 0.89, and 1.25 ± 0.77 U/mg of protein, respectively. Therefore, the AnmK of *R. toruloides* was determined to be the best choice among the six enzymes, and was selected for further work.

### Evaluation of the levoglucosan transport capacity of *S. cerevisiae*

Since the levoglucosan utilization capacity of strain CEN-Rho was still weak, levoglucosan transport was investigated as a limiting step. *S. cerevisiae* strains CEN.PK113-5D and EBY.VW4000 (hxt-null strain) were, respectively, incubated in levoglucosan for 120 min, and the intracellular levoglucosan accumulation of strains was determined. The results showed that CEN.PK113-5D accumulated 3.23 ± 0.00, 3.73 ± 0.01, and 4.02 ± 0.00 mg levoglucosan g DCW^−1^ after 30-, 60-, and 120-min incubation, respectively, while the EBY.VW4000 accumulated 0.00 ± 0.00, 2.92 ± 0.01, and 3.01 ± 0.00 mg levoglucosan g DCW^1^ at the same time points (Additional file 1: Fig. S2).

First, the levoglucosan transport capacity of CEN.PK113-5D, which contained all 18 hexose transporters, was only slightly higher than that of EBY.VW4000, which lacks all 18 hexose transporters. This suggested that the hexose transporters are inefficient transporters for levoglucosan. Second, EBY.VW4000 also accumulated some levoglucosan, suggesting its transporters, such as maltose permease, may have a low capacity to transport levoglucosan. Third, only ~ 4 mg levoglucosan g DCW^−1^ was accumulated in both two strains in the 2-h incubation period, which was only about one-tenth of the accumulation of d-xylose or l-arabinose [30]. These results indicated that levoglucosan transport is a limiting step for levoglucosan utilization in *S. cerevisiae*.

### Screening of Gal2p mutants with improved levoglucosan transport capacity

**Fig. 3:**
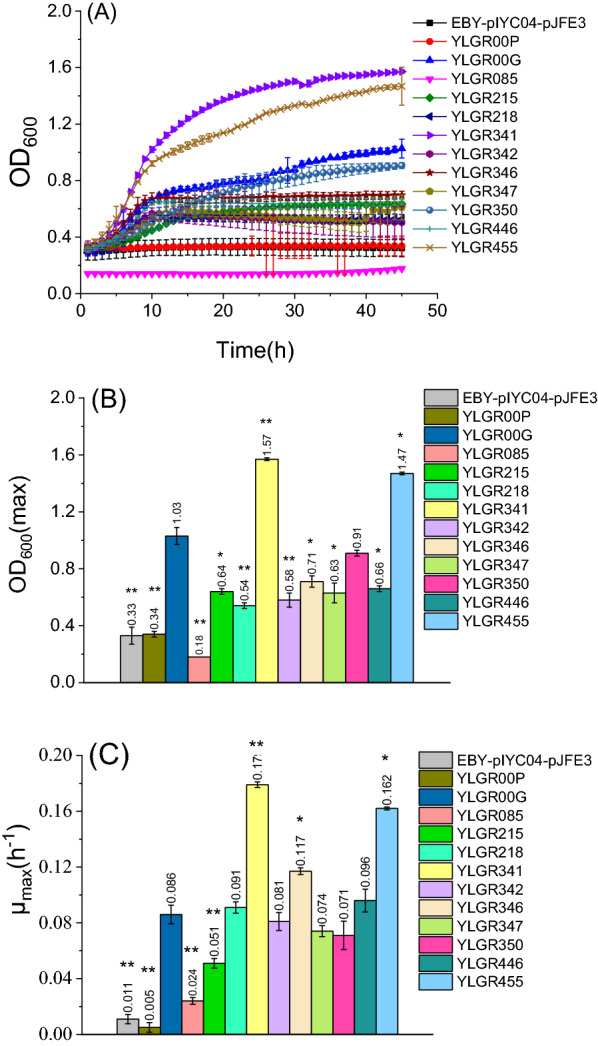
The growth of *S. cerevisiae* strains in the medium using levoglucosan as source carbon source. The pre-cultured strains were transferred to medium SC-URA-HIS supplemented with 5 g L^−1^ levoglucosan. Then the growth of strains was measured by a microplate reader at 30 °C. **A** growth curves; **B** the maximum OD_600_; **C** the maximum specific growth rate (μ_max_). Experiments were performed in triplicate. The data of strain YLGR00G were used as the reference for significance analysis, * p < 0.05; **p < 0.01

Galactose permease Gal2p transporters heavily favor the transport of hexoses and pentoses, such as d-glucose, d-galactose, d-xylose, and l-arabinose [30, 33, 34]. To improve the levoglucosan absorption of *S. cerevisiae*, we used Gal2p as a model to investigate the effects of transport on levoglucosan utilization. The AnmK gene of *R. toruloides* was expressed in EBY.VW4000, which resulted in strain YLGR000. Then the empty vector pJEF3 and recombinant plasmid pJEF3-GAL2^WT^ were, respectively, introduced into YLGR000, resulting in the creation of strains YLGR00P and YLGR00G. The growth rate of the different strains in the medium supplemented with 5 g L^−1^ levoglucosan as sole carbon source (Fig. 3) revealed that the maximum biomass (represented by OD_600_) and the maximum specific growth rate (μ_max_) of strain YLGR00G was 1.03 ± 0.06 and 0.086 ± 0.007 h^−1^, respectively, both of which were much higher than the strain with empty vectors EBY-pIYC04-pJEF3 or YLGR00P. This indicated that Gal2p possesses the capacity to transport levoglucosan, and the overexpression of *GAL2* enhanced the transport and therefore the utilization of levoglucosan.

**Fig. 4:**
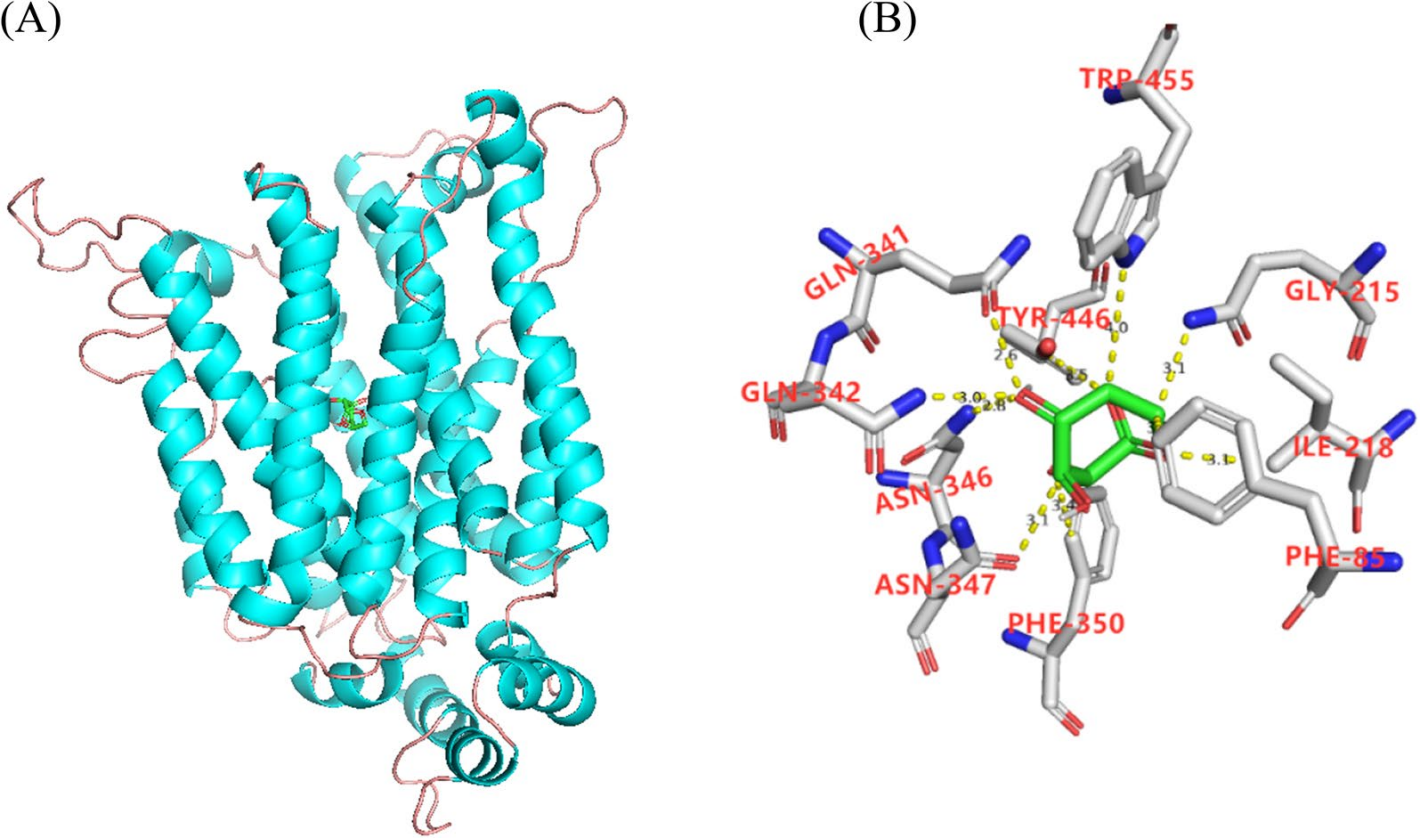
Gal2p docking with levoglucosan model. **A** The full view of Gal2p docking with levoglucosan (green). **B** The amino acid residues (gray) in Gal2p with a distance of < 4 Å from levoglucosan (green)

To improve the levoglucosan transport capacity of Gal2p, a 3D structure of Gal2p docking with levoglucosan was predicted basing on the *E. coli* transporter XylEp [31]. Ten amino acid residues with a distance of  < 4 Å from levoglucosan were selected: F85, Q215, I218, Q341, Q342, N346, N347, F350, Y446, and W455 (Fig. 4). These 10 amino acid residues in plasmid pJFE3-GAL2^WT^, the plasmid containing the wild-type Gal2p, were replaced by the nonpolar residue alanine. The plasmids containing the *GAL2* mutants were introduced into the *S. cerevisiae* strain YLGR000. The resulting strains were named after their mutation sites: for example, the strain YLGR085 expressed the mutant *GAL2*^*F85A*^, strain YLGR215 expressed the mutant *GAL2*^*Q215A*^, etc. Then the levoglucosan transport capacity of Gal2p mutants was evaluated by determining their growth capacity in a 48-well plate with media containing levoglucosan as the sole carbon source. The results (Fig. 3) showed that strains YLRG341 and YLRG455 grew much better than the control strain YLGR00G, which expressed wild-type Gal2p. The μ_max_ of strain YLRG341 and YLRG455 were 0.179 ± 0.002 h^−1^ and 0.162 ± 0.001 h^1^, respectively, indicating an increase of 108% and 88%, respectively, compared to the 0.086 ± 0.007 h^−1^ of YLGR00G. The maximum OD_600_ of strains YLRG341 and YLRG455 were consistently 1.57 ± 0.01 and 1.47 ± 0.0, respectively, an increase of 53% and 45% compared to the 1.03 ± 0.06 of YLGR00G. Together, these results suggested that mutation of Q341A and W455A improved the levoglucosan transport capacity of Gal2p.

**Fig. 5:**
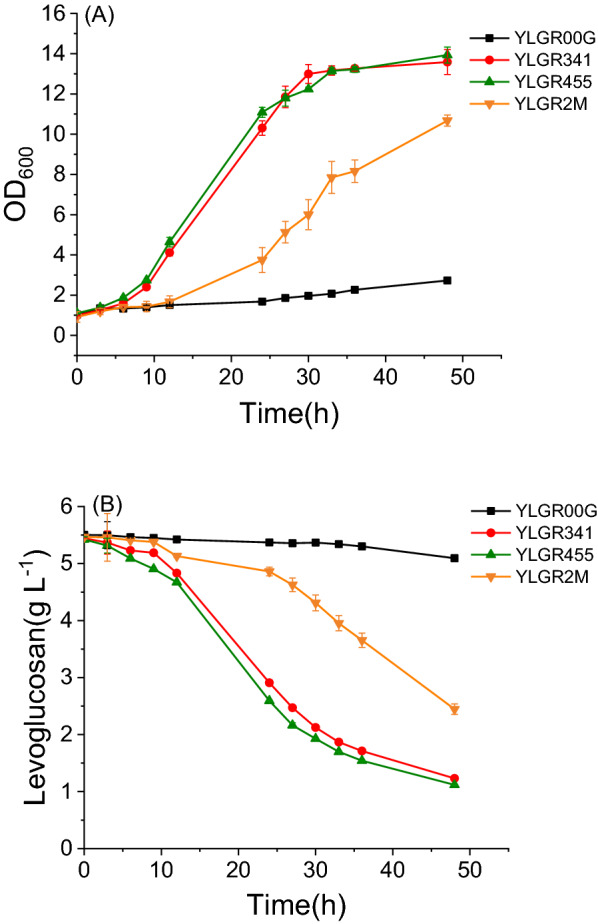
Fermentation profile of strains YLGR00G, YLGR341, YLGR455, and YLGR2M. **A** growth curves; **B** levoglucosan consumption. The cells were cultured at 30 °C, 200 rpm in SC-URA-HIS media supplemented with 5 g L^1^ levoglucosan as a carbon source, the initial OD_600_ was 1.0. Experiments were performed in triplicate

The levoglucosan fermentation profiles of strains YLRG341 and YLRG455 were then investigated in 100-mL shake flasks with 20 mL SC-URA-HIS medium supplemented with 5 g L^−1^ levoglucosan as the carbon source. The initial cell density measured at OD_600_ was 1. The result showed that the growth of strains YLGR341 and YLGR455 was much better than the growth of control strain YLGR00G, although none of them accumulated ethanol, glycerol, or acetate. The specific growth rate (μ_max_) of YLGR341 and YLGR455 was 0.117 ± 0.006 h^−1^ and 0.119 ± 0.003 h^−1^, respectively, while the μ_max_ of YLGR00G was only 0.024 ± 0.001 h^−1^ (Fig. 5A). In consistence with their growth, YLGR341 and YLGR455 consumed 4.21 and 4.31 g L^−1^ levoglucosan in 48 h, respectively, which were 10.4 and 10.7 times the consumption of strain YLGR00G, respectively (Fig. 5B). These results confirmed that transport was one of the limiting steps for levoglucosan utilization of *S. cerevisiae*, and amino acids 341 and 455 of Gal2p were closely related to its levoglucosan transport capacity. Furthermore, the strain YLGR2M, which expressed the combined mutant *GAL2*^*Q341A W455A*^ was constructed. The fermentation result showed that the μ_max_ and consumed levoglucosan of YLGR2M were 0.074 ± 0.014 h^1^ and 3.02 g L^−1^, respectively (Fig. 5), which was lower than that of YLGR341 or YLGR455 but higher than YLGR00G. This indicated that the levoglucosan transport capacity of Gal2p^Q341A W455A^ was higher than Gal2pWT, but lower than Gal2p^Q341A^ or Gal2p^W455A^.

### Q341 and W455 act as barriers to transport of levoglucosan and glucose

**Fig. 6:**
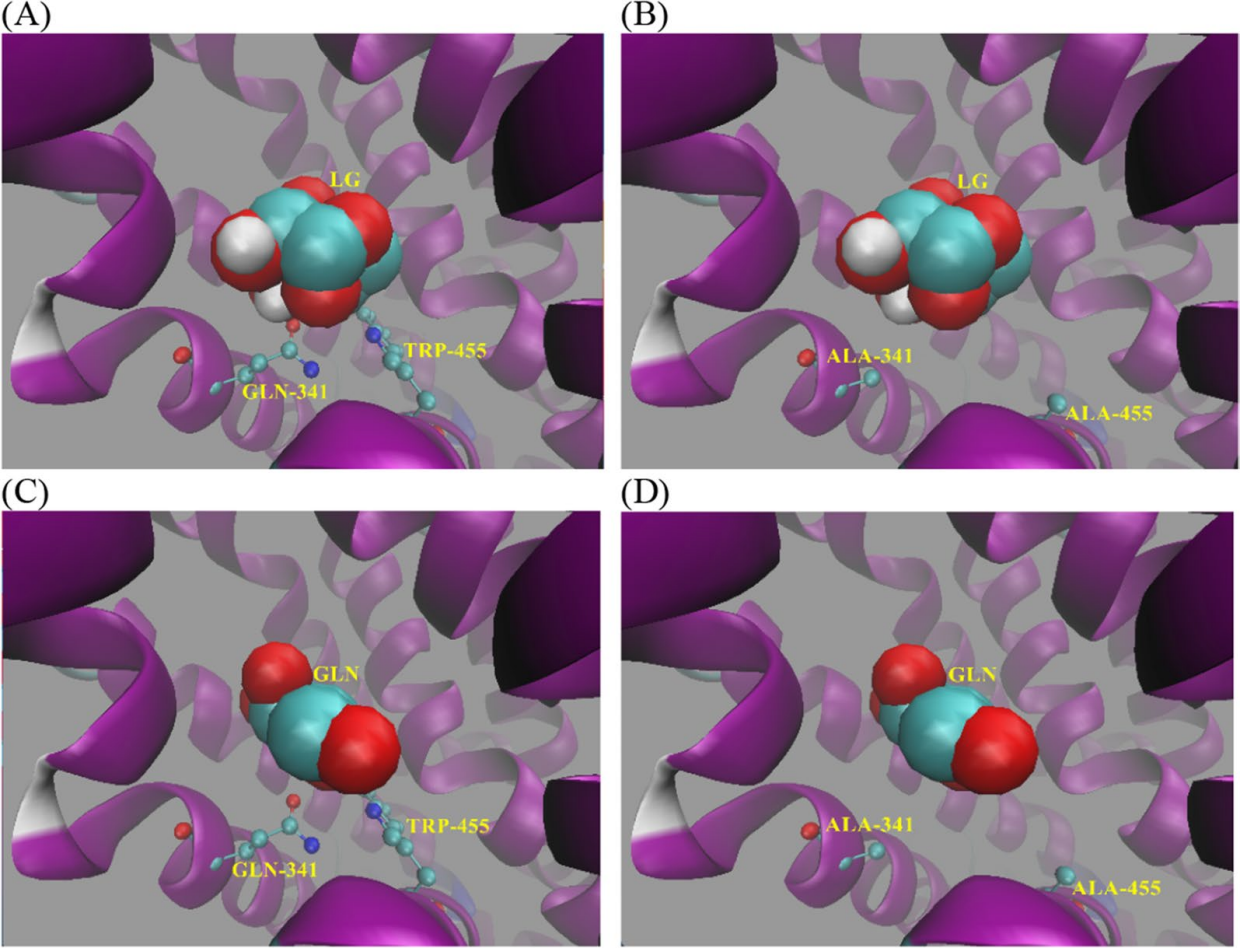
The model of Gal2p and Gal2p^Q341A W455A^ docking with levoglucosan and glucose. **A** Gal2p docking with levoglucosan; **B** Gal2p^Q341A W455A^ docking with levoglucosan; **C** Gal2p docking with glucose; **D** Gal2p^Q341A W455A^ docking with glucose

To reveal how the mutation of Q341A and W455A enhance the levoglucosan transport capacity of Gal2p, the docking state of levoglucosan, as well as glucose, in the Gal2p and Gal2p^Q341A W455A^ was compared (Fig. 6). The model suggested that the amino acid residues Q341 and W455 were located in the center of sugar transport channel and closer to the intracellular side (Fig. 6A), which indicated that they may not contribute to the capture of extracellular glucose or levoglucosan but rather that they act as barriers to the passage of sugars because of their large side chains. Moreover, compared to glucose, which is flat in shape, levoglucosan is thicker because of its ring structure (Fig. 6A and C). It is reasonable to believe that the channel, which glucose can pass through, may be too narrow for levoglucosan. When the large side chain of Q341 or W455 was changed to alanine, which is small, the channel widened (Fig. 6), and levoglucosan could more easily pass through the channel. However, when both the Q341 and W455 were changed to alanine, the channel space may have become too wide to interact with levoglucosan, which may have brought a negative effect on the capture of levoglucosan and led to a lower transport capacity of Gal2p^Q341A W455A^ when compared to Gal2p^Q341A^ or Gal2p^W455A^.

**Fig. 7:**
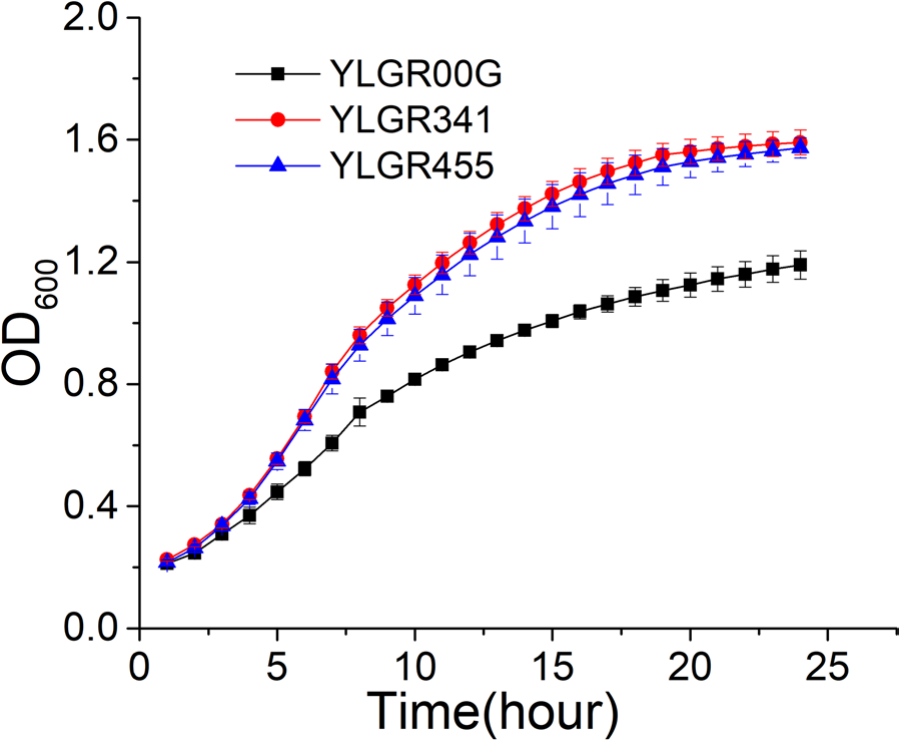
The growth curve of YLGR00G, YLGR341, and YLGR455 using glucose as a carbon source. The cells were cultured in 5 mL SC-URA-HIS medium supplied with 20 g L^1^ glucose for 12 h, then the strains were transferred to fresh medium with the glucose as the carbon source and the growth was measured by a microplate reader at 30 °C. Experiments were performed in triplicate

The Gal2p^Q341A^ and Gal2p^W455A^ docking with glucose model (Fig. 6C and D) suggested that Q341 and W455 were located close to glucose. They may also interact with glucose and play roles in glucose transport capacity. To confirm this hypothesis, the growth of YLGR00G, YLGR341, and YLGR455 in media using glucose as the carbon source was determined by a microplate reader. The results showed that both YLGR341 and YLGR455 grew better than YLGR00G in glucose. The μ_max_ of YLGR341 and YLGR455 were 0.233 ± 0.003 h^1^ and 0.239 ± 0.006 h^1^, respectively, which were 35.5% and 39.0% higher than the control YLGR00G (0.172 ± 0.009 h^−1^) (Fig. 7). These results suggested that Q341 and W455 were also barriers to glucose transport.

## Conclusions

Pyrolysis is a rapid process to saccharify cellulose and obtain levoglucosan. Several microorganism species, such as *E. coli* and *C. glutamicum*, have been engineered to utilize levoglucosan and produce chemicals that are of interest to researchers. However, only a small number of studies have been published on the utilization of levoglucosan in *S. cerevisiae*, which was attributed to lacking efficient levoglucosan kinase. Moreover, the transport of levoglucosan into the cell is also a key limitation step, which is firstly revealed in present work. It points out the strategy to construct levoglucosan-utilizing strains. Furthermore, our engineering strains, which express the AnmK of *R. toruloides* and Gal2p^Q341A^ or Gal2p ^W455A^, consumed ~ 4.2 g L^−1^ levoglucosan in 48 h of fermentation, are the best levoglucosan-utilizing *S. cerevisiae* strains that have been reported. It is necessary to further improve the levoglucosan utilization capacity of strains and introduce the metabolic pathways that produce valuable compounds to make *S. cerevisiae* become another valuable cell factory for the production of compounds using levoglucosan. Mining of more efficient LG transporter and kinase is still a challenge.

## Supplementary Information

**Additional file 1: Figure. S1.** Phylogenetic analysis of LGK ,AnmK and LGK-like proteins. **Figure** **S2.** Intracellular levoglucosan accumulation of CEN.PK113-5D and hxt-null strain EBY.VW4000. CEN.PK113-5D and EBY.VW4000 were cultured in YPD and YPM medium, respectively, for 12 h at 30 °C, then transferred into 20 mL fresh medium for another 12 h cultivation. The cells were then collected by centrifugation, washed twice with ddH_2_O, and resuspended in 30 mL YP medium supplemented with 5 gL^−1^ levoglucosan. After 30 min, 60 min, and 120 min incubation in 30 °C, respectively, cells were harvested and quickly washed with ice-cold ddH_2_O twice, then resuspended with ddH_2_O and placed at 37 °C overnight to extract the intracellular levoglucosan. The accumulation amounts were represented as mg levoglucosan per g dry cell weight (DCW). *, *p*<0.01.

## Data Availability

All data generated and analyzed in this study are included in this published article.
