# Supplementary material for: Both levoglucosan kinase activity and transport capacity limit the utilization of levoglucosan in Saccharomyces cerevisiae
Source: Biotechnol Biofuels Bioprod. 2022 Sep 14;15:94. doi: 10.1186/s13068-022-02195-x (PMC9476349; doi:10.1186/s13068-022-02195-x)
Supplement: Supplementary file 1 — Additional file 1: Figure. S1. Phylogenetic analysis of LGK ,AnmK and LGK-like proteins. Figure S2. Intracellular levoglucosan accumulation of CEN.PK113-5D and hxt-null strain EBY.VW4000. CEN.PK113-5D and EBY.VW4000 were cultured in YPD and YPM medium, respectively, for 12 h at 30 °C, then transferred into 20 mL fresh medium for another 12 h cultivation. The cells were then collected by centrifugation, washed twice with ddH2O, and resuspended in 30 mL YP medium supplemented with 5 gL−1 levoglucosan. After 30 min, 60 min, and 120 min incubation in 30 °C, respectively, cells were harvested and quickly washed with ice-cold ddH2O twice, then resuspended with ddH2O and placed at 37 °C overnight to extract the intracellular levoglucosan. The accumulation amounts were represented as mg levoglucosan per g dry cell weight (DCW). *, p<0.01. [file 13068_2022_2195_MOESM1_ESM.docx]

**Fig. S1** Phylogenetic analysis of LGK ,AnmK and LGK-like proteins.

Numbers show results from bootstrap analyses (1,000 bootstrap replicates).

[1]BAE65712.1; [2]EAU33314.1；[3]CAK44911.1; [4]EAW24693.1 [5]EAL89335.1; [6]EDP50844.1；

[7]EAA59060.1; [8]EED14977.1；

[9]CAP80499.1; [10]EAA32985.1

[11]EAQ84482.1; [12] XP 016276372.1；

[13] ACE79748.1; [14] XP 021869110.1；

[15]EAW22768.1; [16]EAA29125.2

[17]EAW24529.1; [18]EAL86194.1;

[19] EDP50956.1; [20] ABN66269;

[21] EDK40502.2; [22]EDN99772.1;

[23] EAQ93900.1; [24]EAW25579.1;

[25]EAL84290.1; [26] EDP47186.1;

[27] EDU45785.1

[21] hypothetical protein PGUG 04600 of *Meyerozyma guilliermondii*

[1] unnamed protein product of *Aspergillus oryzae* RIB40

[2] conserved hypothetical protein of *Aspergillus terreus* NIH2624

[3] unnamed protein product of *Aspergillus niger*

[4] UPF0075 domain protein of *Aspergillus fischeri* NRRL 181

[5] UPF0075 domain protein of *Aspergillus fumigatus* Af293

[6] UPF0075 domain protein of *Aspergillus fumigatus* A1163

[7] hypothetical protein AN3499.2 of *Aspergillus nidulans* FGSC A4

[8] UPF0075 domain protein of *Talaromyces stipitatus* ATCC 10500

[9] Pc12g08720 of *Penicillium rubens Wisconsin* 54-1255

[10] hypothetical protein of *Neurospora crassa*

[11] conserved hypothetical protein of *Chaetomium globosum* CBS 148.51

[12] anhydro-N-acetylmuramic acid kinase of *Rhodotorula toruloides* NP11

[13] levoglucosan kinase of *Lipomyces starkeyi*

[14] levoglucosan kinase of *Kockovaella imperatae*

[15] UPF0075 domain protein of *Aspergillus fischeri* NRRL 181

[16] UPF0075 domain-containing protein of *Neurospora crassa* OR74A

[17] UPF0075 domain protein of *Aspergillus fischeri* NRRL 181

[18] UPF0075 domain protein of *Aspergillus fumigatus* Af293

[19] UPF0075 domain protein of *Aspergillus fumigatus* A1163

[20] predicted protein of *Scheffersomyces stipitis* CBS 6054

[22] hypothetical protein SS1G 02630 of *Sclerotinia sclerotiorum* 1980 UF-70

[23] hypothetical protein CHGG 02135 of *Chaetomium globosum* CBS 148.51

[24] UPF0075 domain protein of *Aspergillus fischeri* NRRL 181

[25] UPF0075 family protein of *Aspergillus fumigatus* Af293

[26] UPF0075 family protein of *Aspergillus fumigatus* A1163

[27] conserved hypothetical protein of *Pyrenophora tritici-repentis* Pt-1C-BFP

100

100

98

100

98

100

96

92

69

100

100

68

67

63

99

55

98

64

99

85

79

98

52

**Fig. S2** Intracellular levoglucosan accumulation of CEN.PK113-5D and hxt-null strain EBY.VW4000. CEN.PK113-5D and EBY.VW4000 were cultured in YPD and YPM medium, respectively, for 12 hours at 30°C, then transferred into 20 mL fresh medium for another 12 h cultivation. The cells were then collected by centrifugation, washed twice with ddH_2_O, and resuspended in 30 mL YP medium supplemented with 5 g L^-1^ levoglucosan. After 30 min, 60 min, and 120 min incubation in 30°C respectively, cells were harvested and quickly washed with ice-cold ddH_2_O twice, then resuspended with ddH_2_O and placed at 37°C overnight to extract the intracellular levoglucosan. The accumulation amounts were represented as mg levoglucosan per g dry cell weight (DCW). *, p<0.01.

Table S1 Primers used in this study

| **Primers** | **Sequence (5' → 3')** |
| --- | --- |
| G-Rho-up | CTAATCTAAGTTTTAATTACAAAGGATCCTCTAGAGTCGACATGGTTAATGCTTC |
| G-Rho-dn | CTATCGATTTCAATTCAATTCAATCCTGCAGGTCGACTTACCATTTATTATTAAAAAC |
| G-Sch-up | GTTTTAATTACAAAGGATCCTCTAGAGTGGAGAATTCATGGGACTTAACTC |
| G-Sch-dn | GATTTCAATTCAATTCAATCCTGCAGGTCGAGAATTCTTAGGCGTTTCTGTCG |
| G-Mey-up | CTAATCTAAGTTTTAATTACAAAGGATCCTCTAGAGAATTCATGAAAGAGTTGGAC |
| G-Mey-dn | GATCTATCGATTTCAATTCAATTCAATCCTGCAGGAATTCTTACTCGGTGTCAAC |
| G-Lip-up | CATAGCAATCTAATCTAAGTTTTAATTACAAAGGATCGAATTCATGCCAATTGCCAC |
| G-Lip-dn | CAATTCAATTCAATCCTGCAGGTCGACTCTAGAGGATCGAATTCTTAGGCCCAATTG |
| G-Koc-up | CTAATCTAAGTTTTAATTACAAAGGATCCTCTAGAGAATTCATGTTGGACTTCAATATC |
| G-Koc-dn | GATCTATCGATTTCAATTCAATTCAATCCTGCAGGAATTCTTAGGCACCGATTG |
| G-Asp-up | CAATCTAATCTAAGTTTTAATTACAAAGGATCCTCTAGAGAATTCATGGACCCAAACCC |
| G-Asp-dn | CTATCGATTTCAATTCAATTCAATCCTGCAGGAATTCTTACCATTTATTATTAAAC |
| M13F | GTAAAACGACGGCCAGT |
| M13R | CAGGAAACAGCTATGAC |
| pIY-Rho-up | CATCCTTGTAATCCATCGATACTAGTGCGGCCGTCGACTTACCATTTATTATTAAAAAC |
| pIY-Rho-dn | GCATAGCAATCTAATCTAAGTTTTAATTACAAGCGGCCGTCGACATGGTTAATGCTTC |
| GAL2-1-up | GTTTTAATTACAAAGGATCCTCTAGAGTCGAATGGCAGTTGAGGAGAACAATATG |
| GAL2-2-dn | CTATCGATTTCAATTCAATTCAATCCTGCAGGTCGATTATTCTAGCATGGCCTTGTAC |
| F85A-2-up | CCTTGCTTTGTTTGTGTGTTGCCTTCGGCGGCTTCATGGCTGGCTGGGATACCGGTAC |
| F85A-1-dn | GGACAACAAACCCAGAAATAGTACCGGTATCCCAGCCAGCCATGAAGCCGCCGAAGG |
| Q215A-2-up | GCTCCAAAGCACTTGAGAGGCACACTAGTTTCTTGTTATGCTCTGATGATTACTGCAGG |
| Q215A-1-dn | GTACAGTAGCCCAAAAAGATACCTGCAGTAATCATCAGAGCATAACAAGAAACTAGTG |
| I218A-2-up | CTTGAGAGGCACACTAGTTTCTTGTTATCAGCTGATGGCTACTGCAGGTATCTTTTTG |
| I218A-1-dn | CCGTAATTAGTACAGTAGCCCAAAAAGATACCTGCAGTAGCCATCAGCTGATAACAAG |
| Q341A-2-up | GTTGATGGGTGTATTTGTTCAAATGTTCGCTCAATTAACCGGTAACAATTATTTTTTC |
| Q341A-1-dn | GTACCGTAGTAGAAAAAATAATTGTTACCGGTTAATTGAGCGAACATTTGAACAAATAC |
| Q342A-2-up | GTTGATGGGTGTATTTGTTCAAATGTTCCAAGCTTTAACCGGTAACAATTATTTTTTC |
| Q342A-1-dn | CCGTAGTAGAAAAAATAATTGTTACCGGTTAAAGCTTGGAACATTTGAACAAATAC |
| N346A-2-up | GGGTGTATTTGTTCAAATGTTCCAACAATTAACCGGTGCTAATTATTTTTTCTACTAC |
| N346A-1-dn | CGGTACCGTAGTAGAAAAAATAATTAGCACCGGTTAATTGTTGGAAC |
| N347A-2-up | GTGTATTTGTTCAAATGTTCCAACAATTAACCGGTAACGCTTATTTTTTCTACTACGG |
| N347A-1-dn | GACTTGAAAATAACGGTACCGTAGTAGAAAAAATAAGCGTTACCGGTTAATTGTTG |
| F350A-2-up | GTTCCAACAATTAACCGGTAACAATTATTTTGCTTACTACGGTACCGTTATTTTC |
| F350A-1-dn | GGCCAACTGACTTGAAAATAACGGTACCGTAGTAAGCAAAATAATTGTTACCGG |
| Y446A-2-up | CTGTATGATTGTCTTTACCTGTTTTTATATTTTCTGTGCTGCCACAACCTGGGCGCC |
| Y446A-1-dn | GCTGTGATGACCCAGGCAACTGGCGCCCAGGTTGTGGCAGCACAGAAAATATAAAAAC |

**Optimized DNA sequences of synthetic genes**

1、1,6-anhydro-N-acetylmuramic acid kinase from *Rhodotorula toruloides* (*RtANMK*)

ATGGTTAATGCTTCTACTAATGTGAATGGTGCTAATGGTAATGCTAATGGTCATGCTAATGGTGATGCTAATGGTTCTAATGGTGTCAATGGTGCTTCACATGGTGCTCCATTGGACTTCACTGTTTTGGGTTTAAATTCTGGTACTTCTATGGATGGTATTGATTGTGCTTTGTGTAGATTTAGACAAGATTCTCCTGAAGCTCCAATGCATTTTGAATTGTTGAAATATGGTGAAGTTCCACTTCCACAAGGTATTAAGAAAAGAGTTATGAAAATGATTTTGCATAATAGAACTACTCCTGAAGAATTGTCTGAAGTTAATGTTCAATTGGGTGAAACTTTTGCTGATGCTGTTGAATCTTTTATTTCTTCTAATGGTATTGATAGATCTACTATTGATGCTTTGGCTTCTCATGGTCAAACTATTTGGTTGTTGTCTATGCCTGAAGAAGGTCAAGTTAAATCTGCTTTGACTATGGCTGAAGGTTCTTTTTTGGCTTCAAGAACTGGTATTACTTCTATTACTGATTTTAGAATTTCTGATCAAGCTGCTGGTAGACAAGGTGCTCCATTGATAGCATTTTTTGATGCTTTGTTGTTGCATCATCCAACTAAATTGAGAGCTTGTCAAAATATTGGTGGTATTGCTAATGTTTGTTTTATTCCACCTGATCATCAAGGTGGTGTTGATGCTTGTTTTGATTTTGATACTGGTCCTGGTAATGTTTTTATTGATGCTGCTGTTAGATATTTTACTAATGGTGAACAAGAATATGATAGAGATGGTGCTATGGGTAAAAGAGGTAAAGTTAATCAAGCTATGGTTGATAGATTTTTGCAACATAAATATTTTGGTTTGGAACCACCAAAAACTACTGGTAGAGAAGTTTTTAGAGATACTATTGCTCATGATTTGATTAAAGAAGGTGAATCTTTGGGTATGTCTGCTGATGATATTGTTGCTACTGTTACTAGGATTCCTGCTCAAGCTATTGTTGATCATTATAGAAGATATGCTCCATCTCAAGATATTGATGAAATTTTTATGTGTGGTGGTGGTGCTAAGAATCCTAATATTGTTGCTTTCATCCAAGAATCTTACCCAAATACTAAAATTATGATGTTGGATGAAGCTGGTGTTCCTGGTGATGCTAAAGAGGCTTGTACTTTTGCTTGGCAAGGTATGGAGGCTTTGGTTGGTAGATCTATTCCTGTTCCAACTAGAGTTGAAACTAGAAGACCATTTGTTTTGGGTAAAGTTTCTCCTGGTGAAAATTATAGATCTGTTTTGAGAAAAGGTATGGCTTTTGGTGGTGATTCTGATCAATTGCCATGGGTTCATGAAATGGTTAATTATGTTGATGGTAAAGTTTTTAATAATAAATGGTAA

2、1,6-anhydro-N-acetylmuramic acid kinase from *Scheffersomyces stipites* (*SsANMK*)

ATGGGACTTAACTCTGGAACCTCTATCGACGGAATCGACGTTGTTCTTTGTAATTTTAAACAATCTTCTGTTGATAGTCCTTTGCACCTTTCTGTTCTTAAGTACGACGAGATGGATATGCCTCCAGCTCTTAAGAGTAGGGTCTTGAGAATGATCAAGGAGAACAAGACCAAGCTTGAGGAGGTCAGTGAGATTGCCGCCCTTTTGGGTATGGCCTTCGCCAAGGCCGCCGATGATTTCTGCCAGAAGCACGGTATCGAAAAATCTATCATCGATATTATCGGTTCTCACGGACAAACCATCTGGTACGTTCCAGACAGTAAGCCCGGTCAGTGCAGATCTGTCATCACCAGTGGTGAGGCTTGCTACATCGCCGAGAAGATGGGTAAAACCGTCGTCTCTGAGTTTAGAATCTCTGAGCAGAGTGTTGGTAGGCAAGGTGCTCCAATGATCGCCTTCTTCGACAGTTTGCTTCTTGTCCATCCTAAAAAATTTAGGATCTGCCAGAACATTGGTGGAATTGCCAACGTCTGCTTCGTCTTCCCAGAAAAAGACGGAGGTTTGGACAAGTGCTTCGACTACGATACCGGACCCGGTAACGTCTTTATCGACGCCGCCATGAGATACTTCACCAAGGGTACCTTGGAGTATGATAGAGACGGAAAGTGGGGTAAGAGGGGAGTCGTCCACCTTCCACTTGTTGACGAGTTCCTTACCGGTGAGTACTTTTTGAGGGAGCCTCCAAAGACCACCGGTAGGGAGTTGTTCGGAGACTCTGTCGCCTTCGAGCTTATCGAGAATATGATCGCCAAGGGATTGTCTAAGTATGACATTATTGCTACCCTTACCAGAATCACCGCCCAGTCTATCGTCAACGAGTACCATAAGTATTCTAGTGGACATATTGATGAGATCTTCCTTTGCGGTGGTGGAGCCTTGAATCCAAACATCACCGAATATATCCAATCTAGTTTCCCAGACACCAAAATTAATTTGTTGGACGTCACTGGAATTTCTGGTAGTGCCAAGGAATCTATCACCTTCGCCTTCCAAGGTCTTGAGGCTATCTTGGGTAGGAGTTTGATTATTCCAGATAGAGTTGATAGTAGAACCCCAGTTGTTGTCGGAAAGGTCACCCCCGGTAAGAACTATAGGGCCTTGCAGAAAATGGCCGTCGAGTTCACCTCTACTTGTAACTGCGACGGATACTTGCCATCTGTTAGAAAGATGGTCATCGACAGAAACGCCTAA

3、1,6-anhydro-N-acetylmuramic acid kinase from *Meyerozyma guilliermondii* (*MgANMK*)

ATGAAAGAGTTGGACGGTTGCTTGCTTTACTCTCCCGGTTGGCATAGAGTTTTGAGTATCTGTAGTAGTATCACTATGAGTATGAGTGCCGAGAACGGAGCTGCTGAAATCTTGCCAGCCTTCACCCCACCATGCAATGCTAGGTTCGCTACCGAGATGATGAGTGATGACTGCAAGAAAATTTATAAGAGTCCATACTGCTTGTTCCCTCCAGCTATGATCGCTTTCGTCGAGGAGTTGGATGTCAACGTTATGGGTCTTAATTCTGGAACCAGTATCGACGGTATCGACGTCGTCCTTTGTAGATTTAGACAAGCTAGTGTTACCGCCCCACTTCATTTGTCTGTCGTCAAGTACGACGAGATGGAAATGCCAGCCGAGCTTAAAGAGAGGGTCTTGAGGATGATTAAAGAAAATTCTACCTCTCTTGAGGAGGTCTCTCAAATCAGTGCCCTTCTTGGAATGGCCTTCGCCAAGGCCGCCTCTGACTTTTGCACTAAGCATAGTATCGACAAATCTAGTATTGATATTATCGGAAGTCATGGACAGACCATGTGGTATGTCCCAGTCCCAAACCCTAAGAATAACCAATGTAGAAGTGTCATGACCCTTGGAGAGGCTTGCTATATCGCCCAAGAGATGGAGACCACTGTCGTCAGTGATTTTAGAATTAGTGAACAAAGTGTCGGAAGGCAAGGTGCCCCTATGATCGCCTTCTTTGACAGTCTTCTTCTTGTCCACCCAACTAAGTTGAGGGCTTGTCAGAACATCGGAGGTATTGCCAATGTCTGTATCATTTATCCAGAAAACAAAGGTGGTTTGAAGAAGTGCTTCGACTATGACACCGGTCCCGGTAACGTCTTCATCGATGCCGCTATGAGGCACTTCACCAACGGAGAACAAGAATACGACAAAGACGGTGAGTGGGGTAAGCAAGGTGAGGTTGACCAAGAAATGGTTGATGAGTACTTGGCCCAAGAATACTTCCAAAGGCAGCCACCAAAGACCACCGGAAGGGAATACTTTGGTGACCAAGAAGCCTTGAGTCTTATCGAGAAAGGTCTTCAAAAAGGATTGTCTAAATACTCTGTTATCGCTACCTTGACTAGAATTACCGCCCAGAGTATTGTCAACGACTATAGAAGGTACGCTCCCGGTCCAATCGACGAACTTTTCTTGTGCGGTGGTGGAGCCTTCAATCCTAATATTACCGAATTTATTCAATCTAGTTTTCCAGATATGAAACTTATTTTGCTTGACGAGACCGGAATCAGTGGTTCTAGTAAGGAGGCTGTTACCTTCGCCTTCCAAGGTCTTGAGGCTATCTTGGGAAGGCCATTGATCGTCCCAGACAGAGTCGAGTCTAGTACCCCAGTTGTCGTTGGTAAGGTCGCCCCCGGTAAGAACTACAGACATCTTCAAAAACTTTCTTCTCAATTTAGTTTGAGTGTCGGTGACTTGGGTCACCTTCCACCAGTTACCAAGCTTGTTGTTGACACCGAGTAA

4、levoglucosan kinase from *Lipomyces starkeyi* (*LsLGK*)

ATGCCAATTGCCACCAGTACCGGTGATAACGTCTTGGACTTCACCGTCTTGGGTTTGAACAGTGGTACCAGTATGGACGGTATCGACTGCGCTTTGTGCCATTTCTACCAGAAGACCCCAGATGCTCCAATGGAGTTCGAGTTGTTGGAGTACGGTGAAGTCCCTTTGGCTCAGCCTATTAAGCAGAGGGTCATGAGGATGATCCTTGAGGACACCACCTCTCCATCTGAGCTTAGTGAAGTTAACGTTATTTTGGGTGAGCACTTCGCTGACGCCGTCAGACAATTTGCTGCCGAGAGGAATGTTGATCTTAGTACCATCGACGCTATTGCCTCTCACGGTCAGACCATCTGGCTTCTTTCTATGCCAGAGGAGGGTCAAGTTAAATCTGCTTTGACCATGGCCGAAGGTGCCATCTTGGCCAGTAGAACCGGTATCACCAGTATCACCGACTTTAGAATCTCTGATCAAGCCGCCGGAAGACAAGGTGCCCCATTGATTGCCTTCTTCGACGCCTTGCTTCTTCACCACCCTACCAAGCTTAGGGCTTGTCAAAACATCGGTGGTATCGCTAACGTCTGCTTCATCCCACCAGATGTTGACGGTAGAAGGACCGACGAGTACTATGACTTCGACACCGGTCCCGGTAACGTCTTCATCGATGCCGTCGTTAGACACTTTACCAATGGTGAGCAAGAATACGATAAGGACGGAGCCATGGGTAAGAGGGGTAAGGTTGATCAAGAGCTTGTTGATGACTTTTTGAAGATGCCATATTTCCAACTTGATCCACCAAAGACCACCGGTAGAGAGGTTTTCAGAGACACCTTGGCCCACGACTTGATTAGAAGGGCCGAAGCCAAGGGACTTTCTCCAGATGACATCGTCGCTACTACCACTAGAATTACCGCCCAAGCCATCGTTGATCACTATAGGAGATACGCCCCATCTCAAGAAATCGACGAGATCTTCATGTGCGGTGGTGGTGCCTACAATCCAAACATCGTCGAGTTTATTCAACAGTCTTATCCAAATACCAAAATTATGATGTTGGACGAAGCCGGAGTCCCAGCTGGTGCTAAAGAGGCCATCACTTTCGCTTGGCAAGGTATGGAGGCCCTTGTCGGTAGGTCTATCCCAGTTCCAACCAGAGTCGAGACTAGGCAGCACTACGTCTTGGGAAAGGTTTCTCCCGGTCTTAATTACAGATCTGTCATGAAGAAGGGTATGGCCTTCGGTGGAGATGCCCAACAGTTGCCTTGGGTCAGTGAGATGATCGTCAAAAAGAAGGGTAAGGTTATCACTAACAATTGGGCCTAA

5、levoglucosan kinase from *Kockovaella imperatae* (*KiLGK*)

ATGTTGGACTTCAATATCTTGGGTATGAACTCTGGTACCAGTATGGATGGTATCGATTGCGCCTTGTGCCACTTTAGACAAGATCACCCAGATGCTCCAATGCACTTCGAACTTCTTGCCTACGGTGAGGTTCCTCTTGCCCAACCTATTAAAAAGAGAATTATGACTATGATCTTGGAGAATAAAACTTCTCCTAGTGAGCTTTCTGAGGTCAACGTCATCTTGGGAGAGCACTTTGCCGGTGCCGCCCATGAGTTTACCAAGTCTAGGGGTATTTCTTTGGGAGACATCGACGGTATCGCCAGTCACGGTCAGACCATCTGGTTGTTGTCTATGCCAGAGCACGGACAAGTCAAGAGTGCCTTGACCATGGCTGAGGGTGCCATTATCGCTGCTAGAACCGGAATCACCGCTATCACCGACTTTAGAATCTCTGACCAAGCTGCCGGAAGGCAAGGTGCTCCTCTTATCGCCTTCTTCGACAGTTTGTTGTTGCACCACCCAGAAAAGTTGAGGGCTTGTCAAAACATCGGTGGAATTGCTAACGTTTGCTTTATCAAGCCAGACAAGGACGGTAAACTTGACCAAGATGGTTACTACGACTTCGACACTGGACCCGGTAACGTCTTTATCGACGCCGTCATGAGACACTACACCGATGGAAAGGAGGAGTACGATAGAGACGGTTTGTGGGGTAAGAGGGGAAAGGTTGATCAGAAACTTGTTGATGAGTTCTTGCAGAGGCCATACTTCCAGATGGACCCACCTAAGACCACTGGTAGGGAGGTTTTTAGAGATAGTCTTGCCCACGATTTGATCGCCAAGGGAGAGAAGAAGGGATTGAGTCCAGACGACGTCGTTGCTACCGTCACTAGGATCACCGCCCAAGCTATTGTTGACCACTATAGAAGATACGCCCCTAGTCAAGATATCGACGAGATCTACATGTGCGGAGGAGGAGCCTATAACCCTAATATTACTGATTTTATCCAAAAGTGCTACCCACATACTAAGATCATGATGTTGGACGAGGTCGGTGTTCCCGGTGGAGCTAAGGAAGCCATTACCTTCGCTTGGCAAGGTATGGAAGCTCTTGTCGGTAGGAGTATCCCAGTTCCAACCAGAGTCGAGACTAGAAACCCTTTCGTTTTGGGTAAAATTTCTCCCGGTAAAAATTATAGAGATGTCATGAGGAGGGCTATGGCCTTCGGTGCTGGAAGAAGTGAGTTGCCTTGGGTCACCGAGATGGTCTTGAACAAGGGTGGTAAGCCAATCGGTGCCTAA

6、1,6-anhydro-N-acetylmuramic acid kinase from *Aspergillus niger* (*AnANMK*)

ATGGACCCAAACCCATCTGTCAGTAGAAGTTTGGACCTTACCGTCCTTGGTCTTAACTCTGGTACCAGTATGGACGGAATTGACTGCGCCCTTTGCCACTTCCAGCAAGAAACTCCAGACTCTCCTATGAGGTTCGAGTTGCTTAAGTACGGAGAGATCCCACTTGAGCAGACCATTAAAAAGAGAGTTATGAATATTATTCTTCATAATAAAACCTCTCCAAGTGAATTGTCTGAGGTCAACGTTATCTTGGGTGAGACCTTCGCCGCTGCCGTTAAGGAGTTCTGCGGACAATATGACGTTGATATTAGTAGTATCGATGTCATCGGAAGTCACGGACAGACCATCTGGTTGCTTAGTATGCCAGAGGAAGGTGAGGTCAGAAGTGCTCTTACCATGGCCGAGGGATCTTTTATCGCCAGTAGGACCGGTATCACCACCGTCACCGACTTCAGAGTTTCTGACCAAGCTGCCGGAAGACAAGGTGCCCCTTTGATTGCCTTCTTCGACGCCCTTTTGCTTCACCATCCAACCAAGTTGAGGGCTTGTCAGAACATTGGTGGAATCGCCAATGTCTGTTTCATCCCACCAGACAGTTTGGGTGGAACCGACGCTTGCTACGACTTTGACACCGGTCCCGGTAACGTCTTCATCGACGCCGTCGTTAGACACTATACCAATGGTCAGCAAGAATACGACAAGGATGGTGAGATGGGAGCCAGAGGTACCGTTGACCAAGATTTGGTTGATGAGTTCCTTCAGACCCACCCATATTTTAGACTTGACCCACCTAAGACCACCGGTAGGGAGGTTTTTAGAGATACTCTTGCCTTCGACCTTATTAGAAAGGCCGAGTCTAAGGGATTGAGTCCAGATGACGTCGTCGCCACTGTTACCAGAATCACCGCCCAAGCCATTGTTGATCATTATAGAAGGTACGCCCCAAAAGACTTGCCTATCGACGAAATCTTCATGTGCGGAGGAGGTTCTTACAACCCTAACATTACTAGATACATTCAAGCTCACTACCCAGACACCAAGATCTTGATGTTGGACCAAGCTGGTATTCCAGCTAGTGCTAAGGAGGCCATTACTTTCGCTTGGCAAGGTATGGAAGCCGTCGTCGGAAGGAGTATCCCAGTTCCTACCAGAGTCGAAACTAGACAGCCTTACGTCCTTGGTAAAATCTCTCCCGGTAAAAATTATAGGAAAGTCATGCAGCAAGGTATGCAGTTCGGAGGTGACAAACACCACCTTGCCGCTGTTACCGAGTTGGTCAACTACGTCAACGGAAAAGAGTTTAATAATAAATGGTAA
